# Supplementary figures and images for: Heme oxygenase-1 as an important predictor of the severity of COVID-19
Source: PLoS One. 2022 Aug 24;17(8):e0273500. doi: 10.1371/journal.pone.0273500 (PMC9401165; doi:10.1371/journal.pone.0273500)

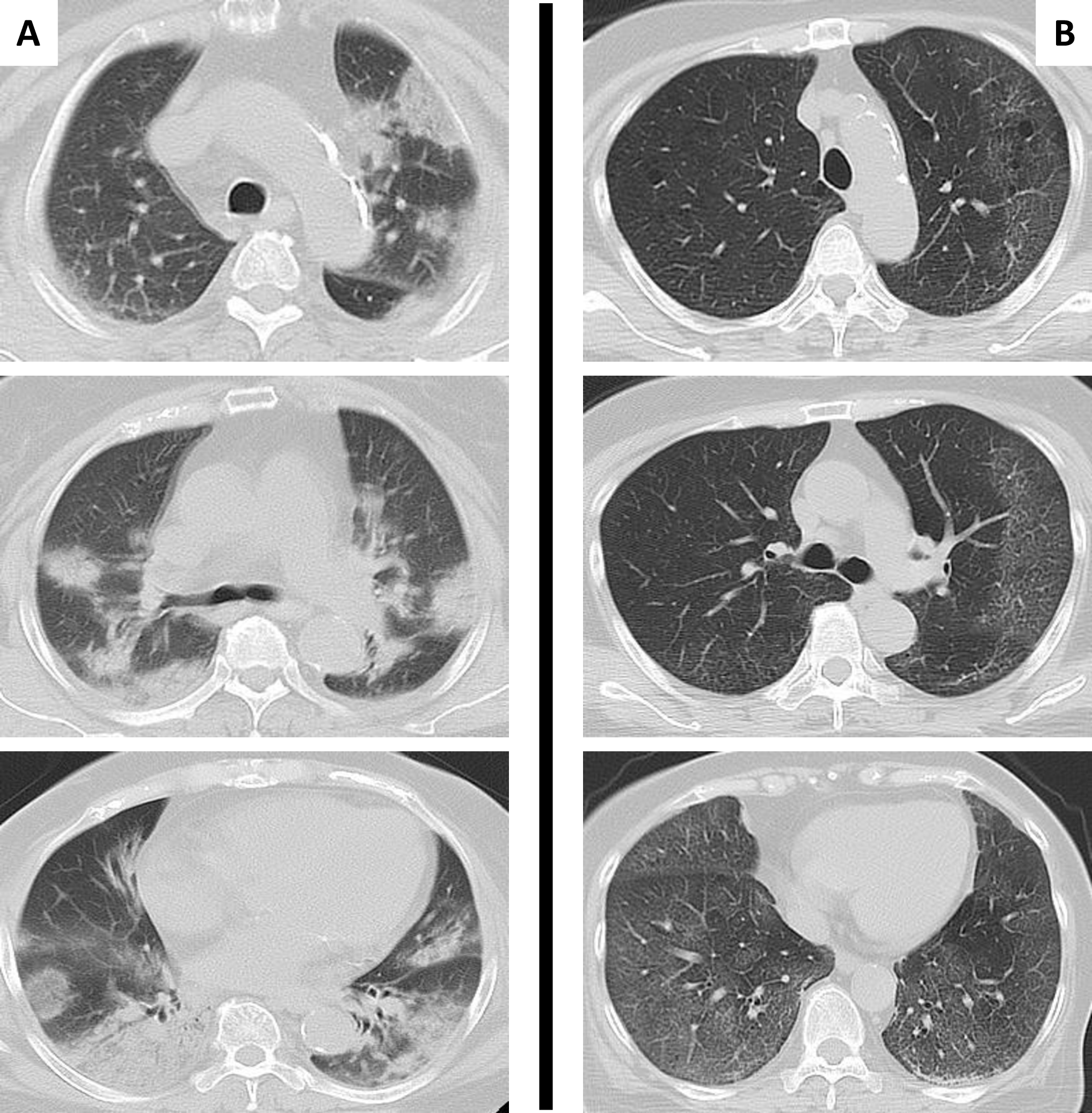

Supplement: S1 Fig — (A) Age 60 years, female. Ground glass opacity (GGO) and consolidation (GGO and consolidation score is 10 points). (B) Age 72 years, female. GGO and reticulation (GGO (without consolidation) and reticular fibrosis scores are 13 points and 1 point, respectively). (TIF) [file pone.0273500.s001.tif]

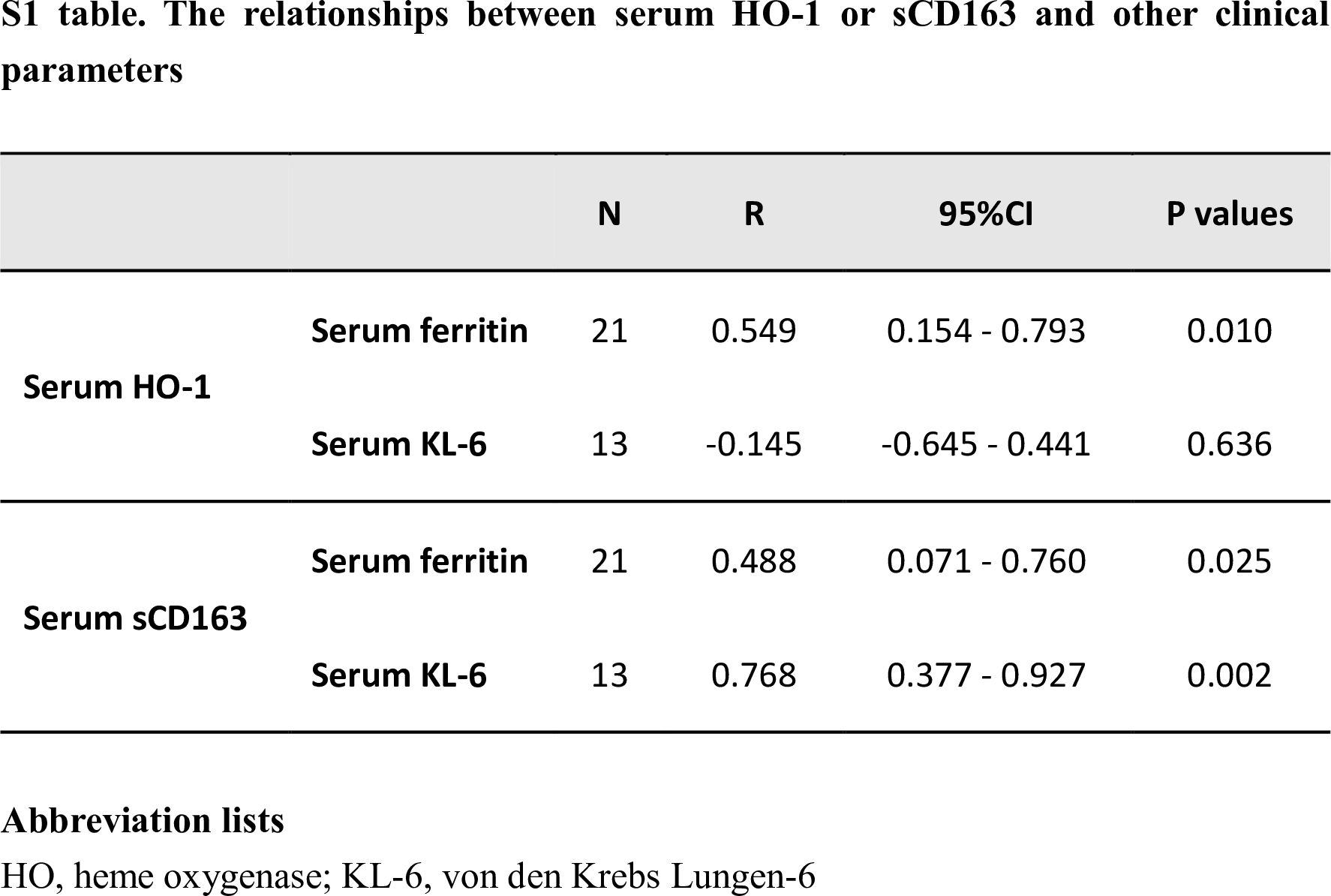

Supplement: S1 Table — (TIF) [file pone.0273500.s002.tif]

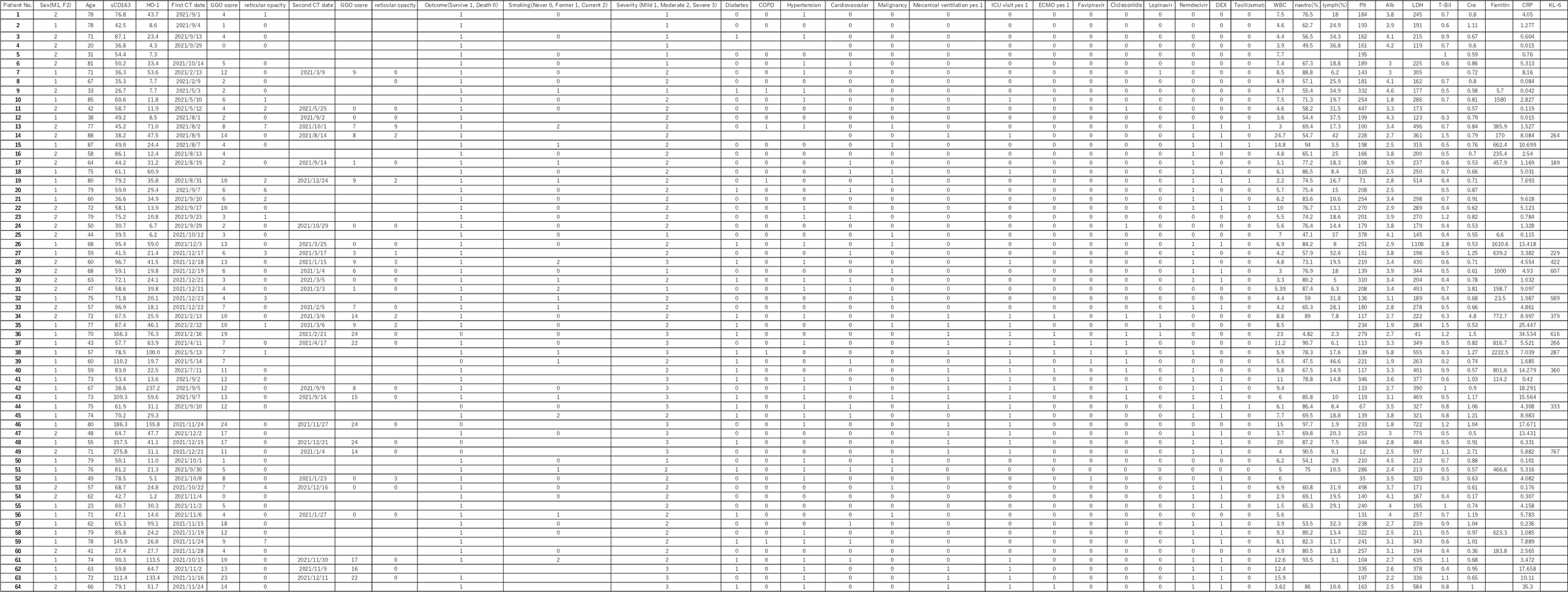

Supplement: S2 Table — (TIF) [file pone.0273500.s003.tif]
