# Supplementary material for: Heme oxygenase-1 as an important predictor of the severity of COVID-19
Source: PLoS One. 2022 Aug 24;17(8):e0273500. doi: 10.1371/journal.pone.0273500 (PMC9401165; doi:10.1371/journal.pone.0273500)
Supplement: Attachment — Submitted filename: Response_to_Reviewers_(PONE-D-21-36509R1).docx [file pone.0273500.s004.docx]

**Response to Reviewers**

**PLOS ONE’s style requirements**

We revised our manuscript according to your style requirements.

**Informed consent**

Concerning informed consent for COVID-19 patients in the present study, the consent was not obtained from each patient because;

1. This retrospective analysis of existing data did not require any interaction with the patient and did not intervene in their treatment.
2. We endeavored to avoid any possibility of SARS-CoV-2 contamination by droplets, airborne particulates, and/or contact infection, which was a mysterious pathogen at the time.

For all patients, consent for participation in this retrospective study was obtained by disclosing that we were conducting a clinical study. We included an opt-out option. We added this description (P12L202-204).

**Statement of financial disclosure**

We did not receive any funding for this study. Therefore, we stated that “The authors received no specific funding for this work.” （P40L600-601）

**Data availability statement**

The minimal data set used and/or analyzed in this current study are shown in the Supporting Information (S2 Table), and are made fully available according to the policy of *PLOS ONE*. We have been clear about the issue of data availability (P40L603-605).

**Supporting information files**

The manuscript was revised according to the Supporting Information guidelines.

**Additional references and description of new contents**

(1) We submitted our manuscript on November 17^th^, 2021. In the meantime, some extremely critical findings associated with a pathogenesis of severe COVID-19 have been published. For example, D. Wendisch *et al*. demonstrated pivotal roles of recruited CD163-expressing M2 alveolar macrophages into lungs from circulating monocytes on the process of COVID-19-induced lung fibrosis (*Cell. 2021 Dec 22*; 184(26): 6243–6261.e27.), which relates to our findings that serum sCD163 showed a strong correlation with serum KL-6, which is widely used as a biomarker for lung fibrosis. Therefore, we edited our manuscript and added certain relevant information including the newest scientific findings. We also added some related references (reference number 39, 46, 47 and 51), and some sentences in ‘discussion’ (P23L312-315 and P24L325-331).

(2) We changed disease name COVID-19 from ‘coronavirus infection disease’ to ‘coronavirus disease’ (P4L69 in ‘Keywords’, P5L76 in ‘Abbreviation lists’, and P6L96 in the ‘Introduction’). We also corrected all expressions of “GGO score” in the original manuscript to “GGO and consolidation score” in this manuscript revision. Also, because ‘HMOX1’ is a gene, we added italics to ‘*HMOX1’* (P8L132, P22L282).

(3) We changed a sentence to be understood more precisely from “since HO-1 is abundantly produced by reticuloendothelial tissues, cytokine storm can be caused by DAD-induced-ARDS in COVID-19.” to “since HO-1 is also abundantly produced by resident macrophages in reticuloendothelial tissues, a remarkable elevation of HO-1 in a cytokine storm can be caused by DAD-induced-ARDS in COVID-19” (P23L301-303).

(4) We reconfirmed the enrolled 64 patients in the present study. Among them, seven were outpatients. We corrected the description to reflect this (P9L147-149, P9L162-165, P10L177-178, P12L209-210).

(5) Blood samples were collected on admission. If sufficient serum samples were not able to be measured, new samples taken on another day were measured. Blood samples were taken from outpatients on their first visit to our hospital. We corrected this: (P10L177-178).

(6) The present version of our entire manuscript was edited, with special attention to grammar and sentence structure.

1. **Reviewers Comments**

**Reviewer #1:1. The authors should include patient inclusion and exclusion criteria in the methods section.**

【Our reply】

Thank you for your suggestions. We noted that the inclusion/exclusion criteria for all COVID-19 patients in the present study were decided by PCR analysis, from nasopharyngeal and pharyngeal swab samples, upon admission to the Yokohama City University Medical Center Hospital (P9L162-165).

**Reviewer #1:2. Authors only reported male sex statistics, how about female sex?**

【Our reply】

Thank you for your comment. We added statistics for female patients on Table 1.

**Reviewer #1:3. Did author check lung function of the patients? It would be great to correlate HO-1 levels with lung function parameters.**

【Our reply】

Thank you for bringing this to our attention. No pulmonary function tests were performed due to our protocols to prevent infection against SARS-CoV-2. We further described this point (P13L222-224).

**Reviewer #2:1. In the methodology, the calculation of the scores is not clear**

【Our reply】

A very important comment, thank you. We added the description of our method for calculating the HRCT scores (P9L152-160). For cases in which HRCT could be re-examined, the scoring was performed again and we examined the relationships between baseline serum HO-1 and the variation of HRCT scores (Figure 4)

**Reviewer #2:2. Are there any particularly interesting data in the deaths and what is the cause of their death?**

【Our reply】

Yes, six patients with severe disease died during their clinical courses, but postmortem analysis was not performed. Therefore, we could provide any details about the cause of their deaths. We described this point (P12L215-216).

**Reviewer #2:3. In this study you suggested that serum HO-1 in COVID-19 may reflect severity or progression to DAD. in the latter case it was rather necessary to study the serological kinetics of HO-1 for at least 6 months**

【Our reply】

A very important comment, thank you. In this study, we could not follow up the serum HO-1 (baseline only). However, we previously experienced an AE involving IPF triggered by COVID-19 in which continuous HO-1 measurements could be clinically useful for tracking the severity of the disease and predicting disease prognosis (reference 51). Therefore, we added the sentences with future direction in the limitation section (P26L362-368).

**Reviewer #2:4. In the methodology you did not specify the time of the serological analysis in relation to the start of the infection.**

**【Our reply】**

Thank you for your question. We measured the serum HO-1 after the diagnosis of COVID-19, but we were unable determine the actual baseline serum HO-1 accurately at the start of infection. In the future, it will be important to evaluate the serological kinetics of serum HO-1 from the initial stage of infection to DAD.

**Reviewer #2:5. The use of the composite prognostic score is a good practice in this study, but it was necessary to calculate these scores on admission and on leaving the hospital for cured patients and also to re-evaluate these scores during the follow-up of these patients**

**【Our reply】**

Agreed. We only measured the serum HO-1 after the diagnosis of COVID-19, but we could not re-evaluate the composite prognostic score including serum HO-1 and HRCT score. However, we could follow up the HRCT scores in 31 patients (46%) and calculated the GGO and consolidation score. We then divided these cases into high and low serum HO-1 groups and found that in the former group, the GGO and consolidation score was significantly decreased (first GGO and consolidation score, 5 (2.3 - 7.8 points); second GGO and consolidation score, 0.5 (0 - 6.8 points) (P = 0.017)) (Fig. 4A), while, in the latter group, it was consistently higher (first GGO and consolidation score, 13 (10 - 17 points); second GGO and consolidation score, 15 (8 - 22 points) (P = 0.828)) (Fig. 4B)　（P20L260-P21L271）. We also added the Figure 4 legend (P39L578-583).

**Reviewer #2:6. Finally it is an interesting study but it remains to first validate the usefulness of the HO-1 assay in COVID-19 pneumonia and also its integration into a multiparametric prognostic score including clinical, radiological and functional data.**

【Our reply】

Yes, we agree. We added sentences in the limitation section (P26L354-358). It is very important to validate the usefulness of the HO-1 assay in COVID-19 in a multi-center prospective study.
